# How Often Are Antibiotic-Resistant Bacteria Said to “Evolve” in the News?

**DOI:** 10.1371/journal.pone.0150396

**Published:** 2016-03-02

**Authors:** Nina Singh, Matthew T. Sit, Deanna M. Chung, Ana A. Lopez, Ranil Weerackoon, Pamela J. Yeh

**Affiliations:** 1 Department of Ecology and Evolutionary Biology, University of California Los Angeles, Los Angeles, California, United States of America; 2 Department of Statistics, University of Nebraska Lincoln, Lincoln, Nebraska, United States of America; Institut National de la Recherche Agronomique, FRANCE

## Abstract

Media plays an important role in informing the general public about scientific ideas. We examine whether the word “evolve,” sometimes considered controversial by the general public, is frequently used in the popular press. Specifically, we ask how often articles discussing antibiotic resistance use the word “evolve” (or its lexemes) as opposed to alternative terms such as “emerge” or “develop.” We chose the topic of antibiotic resistance because it is a medically important issue; bacterial evolution is a central player in human morbidity and mortality. We focused on the most widely-distributed newspapers written in English in the United States, United Kingdom, Canada, India, and Australia. We examined all articles that focused primarily on the evolution of antibiotic resistance, were published in 2014 or earlier, and were accessible in online archives, for a total of 1639 articles. The total years examined per newspaper ranged from 5 to 37 years with a median of 27 years, and the overall range was 1978–2014. We quantified how many articles included the term “evolve” and analyzed how this varied with newspaper, country, and time. We found that an overall rate of 18% of articles used the term “evolve” but with significant variation among countries. Newspapers in the United Kingdom had the highest rate (24%), more than double of those in India (9%), the country with the lowest rate. These frequencies were lower than those found in scientific papers from both evolutionary journals and biomedical journals. There were no statistically significant changes in frequency and no trends when “evolve” usage was compared against variables such as newspaper circulation, liberal/conservative bias, time, and state evolution acceptance in U.S. newspapers. This study highlights the globally low usage of the word “evolve” in the popular press. We suggest this low usage may affect public understanding and acceptance of evolutionary concepts.

## Introduction

Globally, public understanding and acceptance of evolution have been equivocal, with variation across space and time. U.S. polls have consistently shown that a large proportion of Americans do not believe in the evolutionary process [1]. Miller et al.’s [1] paper shows that the percentage of Americans who believe in evolution decreased from 45% to 40% between 1985 and 2005, and the percentage of adults who were not sure whether to accept or deny evolution increased three-fold, from 7% to 21%. In comparison, 80% or more of surveyed adults in Iceland, Denmark, Sweden, and France, and 78% of adults from Japan accept the concept of evolution. Of the surveyed populations, only Turkish adults were more likely than Americans to reject the concept of evolution [1]. However, within both the American and the global scientific community, there is overwhelming acceptance of evolution [2]. This discrepancy means that a large number of people do not believe in a scientific theory widely accepted by scientists, and thus lack what some consider a critical component of scientific literacy [3].

For both the general public and the scientific community, the media plays a noteworthy role in people’s exposure to the term “evolve.” People have reported learning about evolution from a wide array of media sources, including television, museums, and family discussions [4]. Language plays a key role in public understanding of science, and the use of vernacular alternatives for scientific concepts can lead to misunderstandings [5]. In an important and intriguing study, Antonovics et al. [6] randomly chose a subset of 30 scientific papers about antibiotic resistance, half from evolutionary-focused journals and the other half from biomedical journals. They found that papers published in evolutionary-focused journals used the term “evolve” significantly more often than those in biomedical journals. Furthermore, they found that there was a strong correlation between the use of the word “evolve” in scientific papers and its use in corresponding popular press articles.

To our knowledge, there have been no extensive studies to quantify how often “evolve” or its lexemes are used in news articles for the general public. Here we examine some of the most widely circulated newspapers in a number of English-speaking countries and analyze all popular press articles on the subject of antibiotic resistance. We chose antibiotic resistance because it receives a noteworthy amount of media attention as a result of the threat that resistance poses to human health [7], and because it is one of the most well-known topics for which a general audience is able and willing to acknowledge a connection to evolution [8]; among those who have trouble with the theory of evolution, there is less resistance to the idea of bacteria evolving than there is to the idea of humans evolving.

The media can be used to understand the way the public is exposed to the concept of antibiotic resistance. Indeed, a study on the awareness and perception of the drug-resistant bacteria methicillin-resistant *Staphylococcus aureus* (MRSA) found that the media was the most common source of information on MRSA for the majority of patients and hospital visitors [9]. In addition, a 2015 survey by the World Health Organization revealed that levels of knowledge about usage of antibiotics and the threats of resistance were low. While the majority of respondents in the 12 countries included in the survey understood that infections are becoming increasingly resistant to treatment by antibiotics (72%), a majority (76%) also believed that antibiotic resistance is a result of the human body becoming resistant to antibiotics instead of the bacteria, demonstrating a fundamentally flawed understanding of the evolution of antibiotic resistance [10]. While we do not necessarily expect that a better understanding of evolution will reduce the public health threat of antimicrobial resistance, we do think that antimicrobial resistance articles geared towards a general public can be a good gauge of how evolution is described in general audience publications, and by proxy, help us understand how popular-press articles can shape the discourse on a topic such as evolution. Furthermore, we consider this a logical extension and follow-up of the original study on scientific literature [6], where researchers similarly used papers related to antibiotic resistance.

We specifically asked the following:

1. How do popular-press articles compare to scientific papers in the use of the word “evolve”?
2. Are there differences across countries, and over the last four decades, in using the word “evolve”?
3. What is the variation among U.S. newspapers in using “evolve”?

## Materials and Methods

We used web-based resources (Alliance for Audited Media [11], Newspapers Canada [12], OnlineNewspapers.com [13]) to identify the newspapers with the greatest online circulation in English-speaking countries. We focused on the top twenty-five newspapers from the United States (Table 1) and the top two international newspapers from each of several English-speaking countries: *Daily Mail* and *The Guardian* from the United Kingdom, *The Toronto Star* and *The Globe and Mail* from Canada, *The Times of India* and *The Hindustan Times* from India, and *The Age* and *The Australian* from Australia. We searched the exact phrase, “antibiotic resistance” in four UCLA-subscribed electronic databases: Newsbank, LexisNexis, ProQuest, and EBSCOhost. For the U.S. newspapers, we excluded *The Boston Globe*, *The Chicago Tribune*, and *The Detroit Free Press* (3 newspapers excluded, 22 U.S. newspapers total) because we were unable to obtain full access to most or all of their articles. We identified the U.S. newspapers as “liberal” or “conservative” based on previous literature [14] and used the online database that the literature cited, Mondo [15], to classify newspapers that had not previously been classified in [14]. We further categorized the U.S. newspapers by state views on evolution [16], presence of a science section [17], circulation [11] (S3 Table), and city population sizes [18].

**Table 1:** Number of Relevant Articles Examined for Each Newspaper.

Newspaper	Number of Relevant Articles on Antibiotic Resistance
Chicago Sun Times (Illinois, United States)	26
Cleveland Plain Dealer (Ohio, United States)	34
Dallas Morning News (Texas, United States)	45
Denver Post (Colorado, United States)	36
Honolulu Star Advertiser (Hawaii, United States)	2
Houston Chronicle (Texas, United States)	71
Los Angeles Investor's Business Daily (California, United States)	3
Los Angeles Times (California, United States)	186
Miami Herald (Florida, United States)	41
Minneapolis Star Tribune (Minnesota, United States)	18
New York Daily News (New York, United States)	7
New York Post (New York, United States)	2
New York Times (New York, United States)	122
Newark Star Ledger (New Jersey, United States)	59
Newsday (New York, United States)	1
Philadelphia Inquirer (Pennsylvania, United States)	51
Riverside County Press Enterprise (California, United States)	10
Salt Lake City Deseret News (Utah, United States)	68
San Francisco Chronicle (California, United States)	34
St. Paul Pioneer Press (Minnesota, United States)	53
USA Today (Virginia, United States)	78
Wall Street Journal (New York, United States)	96
Daily Mail (United Kingdom)	144
The Guardian (United Kingdom)	58
The Globe and Mail (Canada)	136
Toronto Star (Canada)	79
The Hindustan Times (India)	24
The Times of India (India)	86
The Age (Australia)	24
The Australian (Australia)	45

An article was considered relevant if there was a direct focus on and discussion of antibiotic resistance. Letters to the editor, articles with multiple disconnected topics unrelated to antibiotic resistance, articles that were less than 100 words, and repeated articles were excluded.

We carefully read each article published in 2014 or earlier. To ensure relevance, only articles that directly discussed antibiotic resistance were included in the final databases (S1 Table). We included articles that were written by authors from other newspapers because the newspaper we were examining published the aforementioned article, even if it was not their own original content. Articles that were less than 100 words were not counted because we considered that length too short to extensively cover antibiotic resistance. Letters to the editor, articles containing multiple disconnected topics that were not related to antibiotic resistance, and duplicate articles were also excluded. In the case of duplicate articles, we chose the longer article (some were republished with extra material) or the more recent article if they were identical.

We searched for the word “evolve” (and its lexemes) using the internet browser search feature by typing in “evol” and carefully reading the paragraph to ensure that the word was used in the context of evolution of antibiotic resistance. Furthermore, we also used the search feature to quantify the use of a number of words that were often used instead of or in addition to “evolve,” including “rise,” “emerge,” “develop,” “increase,” and “mutate” (and their lexemes). These words were chosen based on a preliminary survey of the articles. When analyzing the data for country comparisons, we only used the top two newspapers by circulation from the United States (*The New York Times* and *The Wall Street Journal*), but we used all of the U.S. newspapers for results only concerning the U.S. and to find the total percentages of usage for each word.

We performed a qualitative analysis on 33% of all the articles from S1 Table to determine how articles used the word “evolve” and other substitute words (S2 Table). We used a random sequence generator to generate 541 articles, and then examined the context in which each word was presented. We examined contextual evidence in the form of sentences or phrases to present a clear indication of the author’s point about the evolution of antibiotic resistant bacteria. We also compared the frequency of “evolve” usage for each U.S. newspaper to the newspaper variables described above (S3 Table).

We examined if the frequency of “evolve” usage differed based on the paper’s political orientation or whether the paper contained a special section reserved for science. Since a publication’s political orientation and scientific aspect are categorical explanatory variables and the usage of “evolve” can be considered a binomial response variable, we utilized odds ratios to determine if the odds of seeing “evolve” was significant based on the aforementioned explanatory variables.

We further assessed whether the usage of “evolve” changed based on the city’s population and over time. Since both year and population size are quantitative predictor variables, we used a binomial regression model to ascertain if a significant relationship existed based on the binomial distribution of the response variable.

## Results

**Fig 1:**
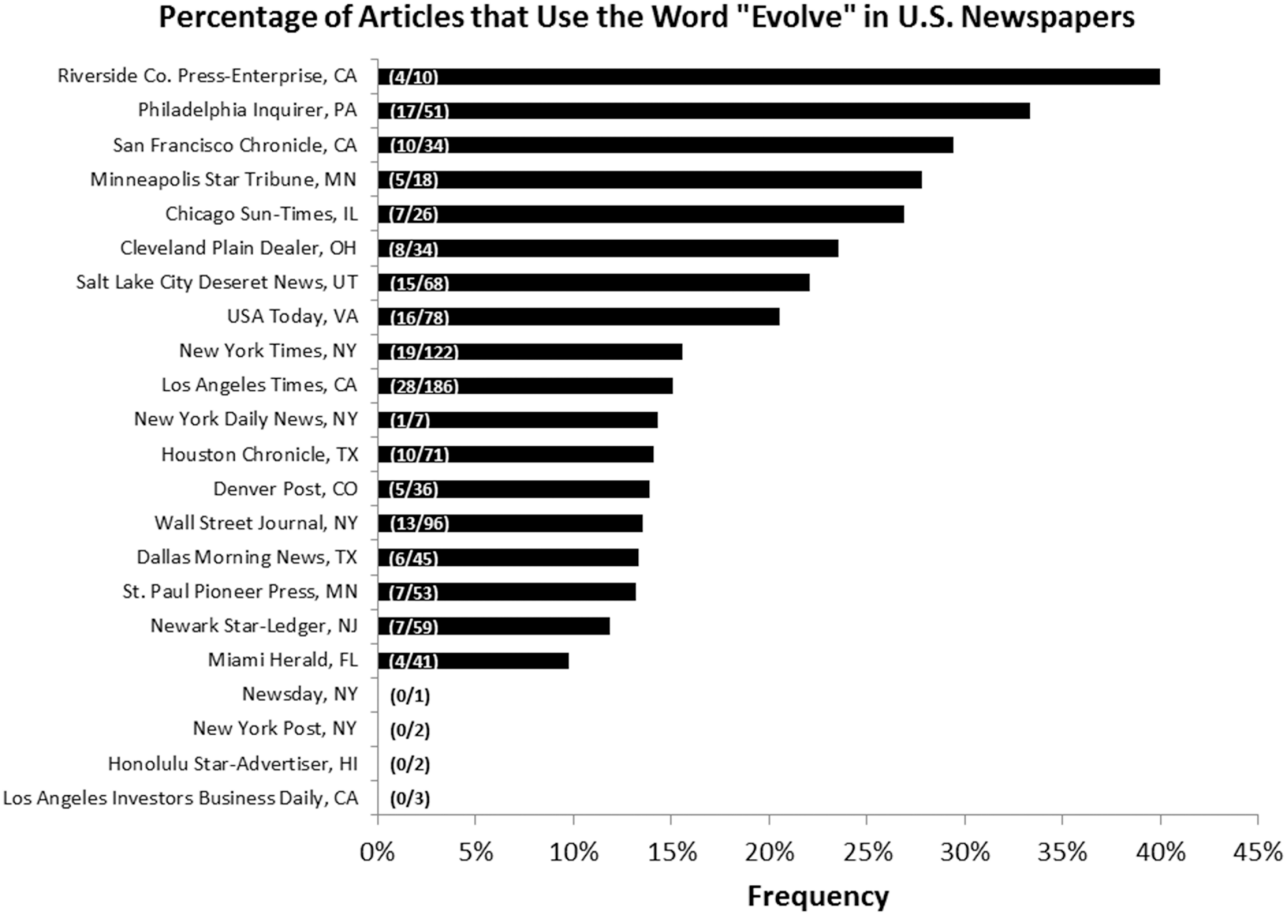
Percentage of US Newspapers Using “Evolve.” Parenthetical bar labels indicate: (number of relevant articles using “evolve” / number of relevant articles examined).

**Fig 2:**
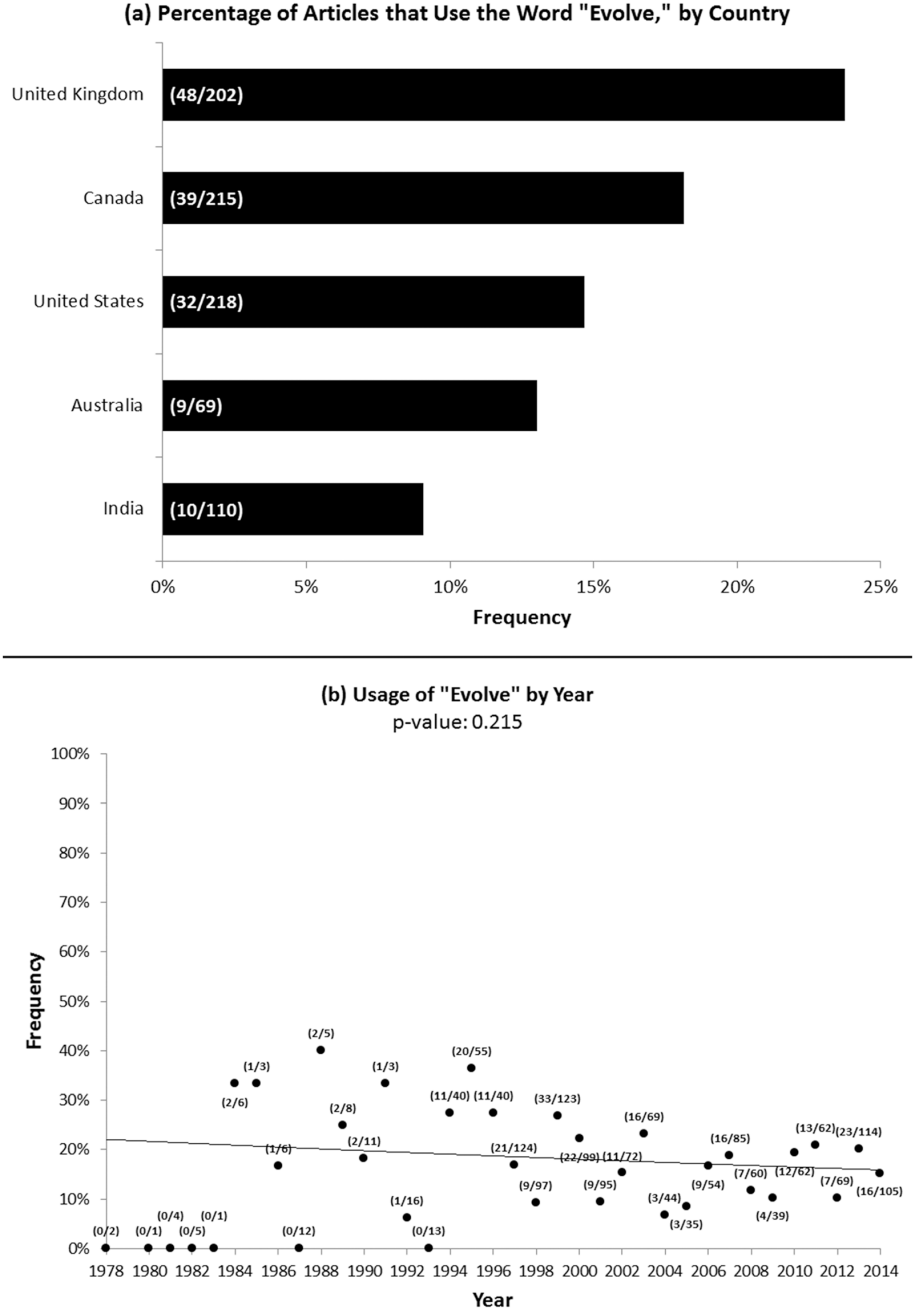
Percentage of Newspapers Using “Evolve” by Country and by Year. (a) Frequencies that the word “evolve” is used for the top two newspapers by circulation in each country, and (b) Frequencies that the word “evolve” is used each year for all relevant articles examined. Parenthetical data labels indicate: (number of relevant articles using “evolve” / number of relevant articles examined). Years without data points indicate that no relevant articles were examined from that year.

We found that 18% of all the articles we examined used the word “evolve” when discussing antibiotic resistance. Within the U.S., the percentage of articles using “evolve” within a given newspaper ranged from 0% to 40% (Fig 1), and there was large variation in the number of relevant articles about antibiotic resistance among the top 25 U.S. newspapers we could access fully through UCLA archives (1–186, with a median of 45) (Table 1). Across all countries, the percentage of popular press articles using “evolve” ranged from 9% (India) to 24% (U.K.) (Fig 2a). Over the time range we examined, there were no statistically significant trends regarding the overall use of “evolve” over time (p-value: 0.215) (Fig 2b) or the use of “evolve” by country over time (S1 Fig).

**Fig 3:**
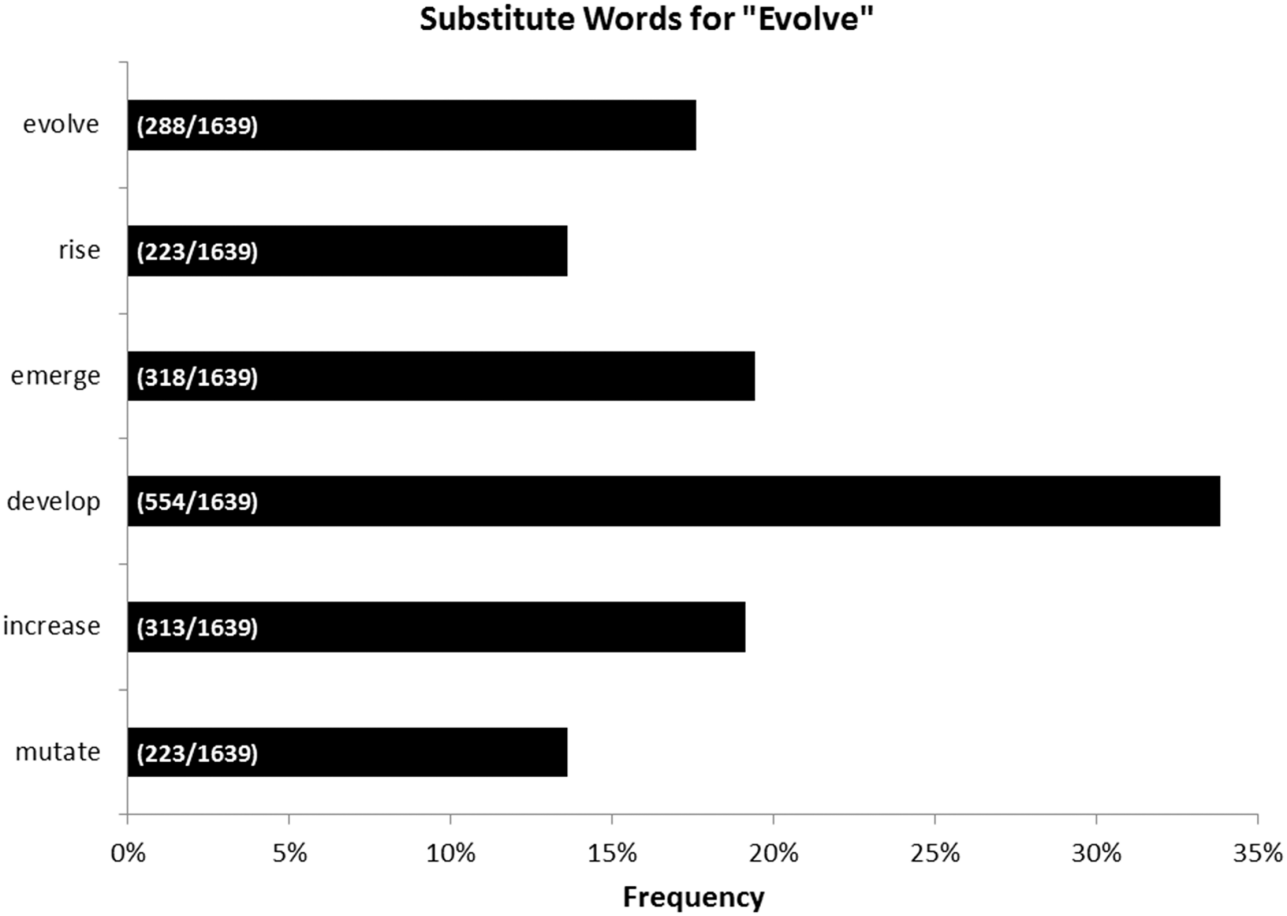
Substitute Words for “Evolve.” Frequencies that the word “evolve” or substitute words for “evolve” are used for all relevant newspapers examined. Parenthetical bar labels indicate: (number of relevant articles using the indicated word / number of relevant articles examined).

A wide range of words were used instead of “evolve,” including “rise,” “emerge,” “develop,” increase,” and “mutate.” We found that “evolve” was used least frequently among all replacement words with the exception of “rise,” even though “evolve” was the scientifically appropriate word choice in all of these situations (Fig 3). There was no relationship between the frequency of use of “evolve” and the populations of the cities that the U.S. newspapers were based in (p-value: 0.160) (Fig A in S2 Fig). There was a positive correlation between the absolute numbers of articles about antibiotic resistance and populations of the cities (p-value: 0.024; r^2^: 0.278) (Fig B in S2 Fig), although this does not correct for the overall numbers of articles a given newspaper publishes. Note that the significant result is largely due to the presence of the two largest cities (New York City and Los Angeles); if the two newspapers with the greatest circulations from both cities (*The Wall Street Journal* and *The Los Angeles Times*) were removed, we would no longer have significance (p-value: 0.872, r^2^ = 0.002).

We examined the relationship between the frequency of use of “evolve” and other variables in U.S. newspapers, including: political orientation (i.e. liberal or conservative leaning), state views on evolution [16], presence of a science section [17], and circulation [11], but did not find any significant trends (S3 Table). Specifically, there was no correlation between the usage of “evolve” and circulation nor between “evolve” and state views of evolution (p-values: 0.870 and 0.565, respectively). With regards to a newspaper’s political orientation, we found no significant odds for an article using “evolve” in a conservative newspaper as compared to a liberal newspaper (95% Confidence Interval for odds ratio: (0.704, 1.543)). While the estimated odds of using “evolve” were 1.33 times as large for science sections as compared to health sections, this did not prove to be significant (95% Confidence Interval for odds ratio: (0.887, 1.99)).

## Discussion

Our findings show that popular press articles on antibiotic resistance mention the word “evolve” at a much lower rate than scientific papers in both evolutionary journals and biomedical journals (18% in newspapers versus 100% in evolutionary journals and 33% in biomedical journals [6]). There were significant differences among newspapers in the United States, as well as among different countries. Within the U.S., it was not surprising to see that newspapers in larger metropolitan areas (as displayed in the U.S. 2010 Census Bureau [18]) tended to have a higher number of articles on antibiotic resistance, perhaps because many of these newspapers have their own science sections and science staff. While one might expect to find a correlation between city size and frequency of newspaper usage of the word “evolve,” based on findings that larger cities tend to have a more educated population [19], we failed to find any correlation between the two. However, this may be due to the fact that our newspaper selections did not reflect a random sample of newspapers across the country. Our failure to find any correlation with the other variables we examined in U.S. newspapers (S3 Table) indicates that lack of usage of “evolve” is not confined to certain types of publications.

Globally, a recent poll published in 2010 found that public acceptance of evolution was 55% in the United Kingdom, 51% in Australia, 45% in Canada, 39% in India, and 28% in the United States [20]. This poll is somewhat consistent with our findings; newspapers from the United Kingdom and Canada used the word “evolve” at greater frequencies, 24% and 18%, respectively. However, for India we found that only 9% of relevant newspaper articles used the word “evolve,” and only 13% of articles did so for Australia. This is in comparison to 15% in the top two American newspapers, even though the poll indicates greater acceptance of evolution in Australia and India than in the United States. The high public acceptance of evolution in the United Kingdom may be due in part to the fact that evolution and Darwin originated in the U.K. Ideas are sometimes better accepted in the region or country in which they originated, similar to the ideas of consumer ethnocentrism, country-of-origin effects, and the bias for domestic products over foreign ones [21–24].

The widespread public rejection of evolution [20] is problematic because it can affect progress and policy in areas such as medicine, the environment, and public health [25]. Public confusion about evolution is exacerbated by euphemisms used by the press when describing topics such as antibiotic resistance. Marlene Zuk’s popular press article sums up the situation perfectly when she writes, “Doctors need Darwin, and the media has to stop using vague terminology that makes it sound as if bacteria were suddenly, inexplicably motivated to deter penicillin through spite” [26]. By downplaying the role of evolution in critical medical issues such as antibiotic resistance, we may also be downplaying the magnitude of the issues at hand. Antibiotic resistance does not simply “emerge” or “develop;” it “evolves,” often due to human misuse of antibiotics [27]. Some articles used substitute words to complement “evolve” by discussing more specific aspects of the evolution of antibiotic resistance (e.g. the random mutation that plays a part in it) (S2 Table). Indeed, just using the word “evolve” in an article could be considered a fairly low bar regarding scientific content. While some articles did use “evolve,” most of the articles that use the word “evolve” failed to differentiate between the mutations that arise randomly and the selection pressures that favor some mutations over others. Thus, even articles that do mention evolution may be explaining evolutionary concepts without much detail, or worse, incorrectly.

When examining the reasons why journalists demonstrate a preference for alternative words such as “develop” instead of “evolve,” we can imagine several possibilities. First, it is possible that journalists care more about bringing antibiotic resistance to public attention than depicting it accurately. Evolution is often perceived as a slow process of gradual change and words like “develop” and “mutate” are perceived as occurring faster, thus making them more exciting and threatening [6]. Previous corpus linguistics studies on antibiotic resistance have discovered that the “race,” “war,” and “apocalypse” metaphors are often invoked to discuss the topic in the media [28, 29]. That is, antibiotic resistance is discussed as a race in which bacteria are staying one step ahead of scientists [28].

Some articles use “mutate” as a supplement to evolution. For example, after stating that “resistance to antibiotics is a growing problem, caused by the devilish ability of microbes to evolve new defenses against drugs,” one article followed with: “a chance mutation can give a microbe resistance” (Table A line 9 in S2 Table). However, many more articles, like one titled, “Mutant bacteria running amok in suburbs” use “mutate” to support a catastrophe discourse [29].

In addition, “emerge” is often used as a substitute for “evolution.” Antonovics [6] speculated that this substitution was a “simplified phraseology” that has spread merely through recurrent usage. There are differing perspectives on whether to use “emerge” or “evolution,” considering that “emergence” potentially invokes mechanisms of evolution such as mutation and recombination [6].

A second reason that writers may not use “evolve” is that scientists and journalists both may adjust their writing style to garner favor with politicians who often play a role in determining what research gets funding and attention [29]. Thus, an extension of this idea is that journalists may avoid the word “evolve” in order to not offend those who do not believe in evolution, and therefore appeal to a wider audience [29].

The role of media in disseminating scientific information is complex [28]. Scientists often believe that the understanding that the public gains from news coverage shapes their viewpoints toward popular scientific consensus [30] and leads to a lack of controversy [30]. The traditional “deficit model” argues that the difference in science understanding between experts and the public is due to public ignorance of science, and can thus be easily remedied by providing the public with science knowledge [30]. However, this has been widely challenged, as various factors, such as the public’s interactions with their communities and their personal biases complicate the science-society relationship [30]. Still, the media plays an important role in framing the public debate on evolution, providing the general public with the common language used in discourse on the topic.

Because antibiotic resistance is strongly affected by human actions, including within hospital, local, and national governmental policy, there is further motivation for the media to inform the public well. The World Health Organization’s “Antibiotic Resistance: Multi-Country Public Health Survey,” which showed low levels of public understanding of antibiotic resistance, also highlighted the dangerous antibiotic usage habits of the survey respondents. Among those surveyed, 32% believed they should stop taking antibiotics when they felt better and 64% thought that conditions such as the cold and flu could be treated with antibiotics. Explaining antibiotic resistance by using the correct terminology is key both to helping the public reduce the threat of resistance and in promoting acceptance of evolution [10]. Low usage of the word “evolve” by the popular press in discussing antibiotic resistance roughly correlates with low levels of evolution acceptance within individual countries. This suggests that exposure to this key word could promote public acceptance of evolution. However, we acknowledge that this is likely not a universally accepted goal. Because evolution can be a controversial topic, the approach adopted by widely consumed media might have significant implications for the way the public perceives evolution [31]. Given that media outlets are one important source of scientific information for people globally, it is important that the media use the term “evolve” when it is appropriate.

## Supporting Information

S1 Fig Frequencies of “Evolve” by Year for Each Country. Frequencies that the word “evolve” is used for all examined newspapers by year (a) in the United States, (b) in the United Kingdom, (c) in Canada, (d) in India, and (e) in Australia. Parenthetical bar labels indicate: (number of relevant articles using “evolve” / number of relevant articles examined). Years without data points indicate that no relevant articles were examined from that year.
(TIF)

S2 Fig U.S. City Population vs. Usage of “Evolve” and Number of Relevant Articles. (a) Correlation between city population (according to 2010 U.S. Census Bureau) and frequencies that the word “evolve” is used for the most widely circulated newspaper examined of each U.S. city represented by our study. The outlier in the x direction, representing the newspaper with the greatest circulation from New York (*The Wall Street Journal*) was excluded from this chart and regression. (b) Correlation between city population (according to 2010 U.S. Census Bureau) and number of relevant articles identified for the most widely circulated newspaper examined of each city represented by our study.
(TIF)

S1 Table List of Articles Used in Analysis. (XLSX)

S2 Table Usage of “Evolve” and Substitute Words in Random Sample. Approximately one-third of all the articles were randomly chosen for qualitative analysis. The context of each substitute word in the various articles is given, arranged by (a) article and by (b) word. “Evolve” phrases are in green, “mutate” phrases are in red, “develop” phrases are in purple, “emerge” phrases are in blue, “rise” words are in orange, and “increase” words are in brown.
(XLSX)

S3 Table Analysis of Trends in U.S. Newspapers between Usage of “Evolve” and Other Variables. Lists each U.S. newspaper with its percentage of articles using “evolve,” state view on evolution, presence of a science section (or closest alternative such as a health section), political orientation (liberal, conservative, or no bias), and circulation. The 95% Confidence Intervals for Odds Ratios or P-Values used to analyze trends between usage of “evolve” and these variables are provided.
(PDF)
