# Supplementary figures and images for: How Often Are Antibiotic-Resistant Bacteria Said to “Evolve” in the News?
Source: PLoS One. 2016 Mar 2;11(3):e0150396. doi: 10.1371/journal.pone.0150396 (PMC4775048; doi:10.1371/journal.pone.0150396)

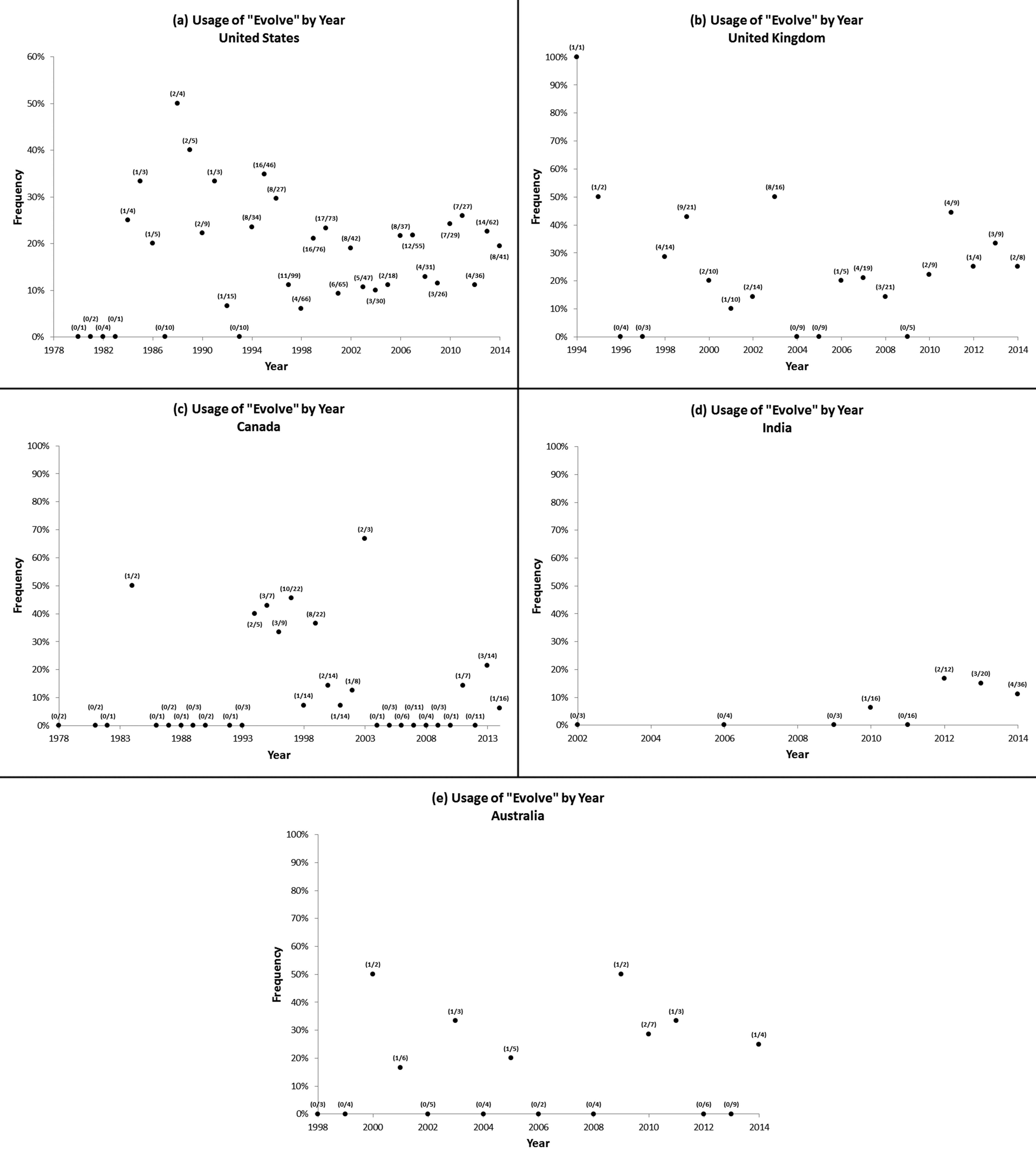

Supplement: S1 Fig — Frequencies that the word “evolve” is used for all examined newspapers by year (a) in the United States, (b) in the United Kingdom, (c) in Canada, (d) in India, and (e) in Australia. Parenthetical bar labels indicate: (number of relevant articles using “evolve” / number of relevant articles examined). Years without data points indicate that no relevant articles were examined from that year. (TIF) [file pone.0150396.s001.tif]

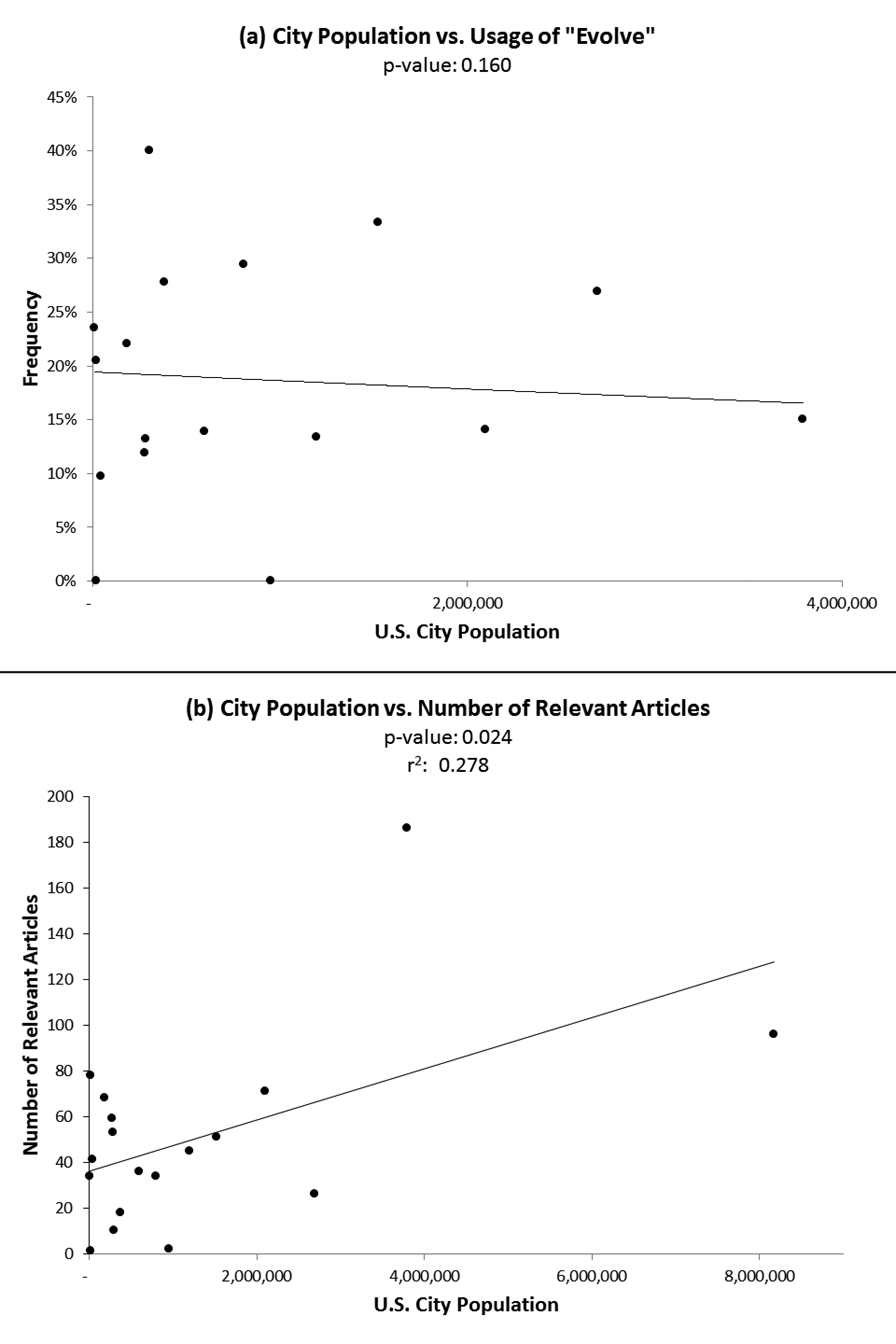

Supplement: S2 Fig — (a) Correlation between city population (according to 2010 U.S. Census Bureau) and frequencies that the word “evolve” is used for the most widely circulated newspaper examined of each U.S. city represented by our study. The outlier in the x direction, representing the newspaper with the greatest circulation from New York (The Wall Street Journal) was excluded from this chart and regression. (b) Correlation between city population (according to 2010 U.S. Census Bureau) and number of relevant articles identified for the most widely circulated newspaper examined of each city represented by our study. (TIF) [file pone.0150396.s002.tif]
