# Supplementary material for: How Often Are Antibiotic-Resistant Bacteria Said to “Evolve” in the News?
Source: PLoS One. 2016 Mar 2;11(3):e0150396. doi: 10.1371/journal.pone.0150396 (PMC4775048; doi:10.1371/journal.pone.0150396)
Supplement: S3 Table — Lists each U.S. newspaper with its percentage of articles using “evolve,” state view on evolution, presence of a science section (or closest alternative such as a health section), political orientation (liberal, conservative, or no bias), and circulation. The 95% Confidence Intervals for Odds Ratios or P-Values used to analyze trends between usage of “evolve” and these variables are provided. (PDF) [file pone.0150396.s005.pdf]

S3 Table.
Analysis of Trends in U.S. Newspapers between Usage of “Evolve” and Other Variables.

U.S. Newspaper Classification

Title	% Articles "Evolve"	State View on Evolution	Science Section?	Political Orientation	Circulation
Chicago Sun-Times (IL)	27%	37%	Health	No bias	470,548
Cleveland Plain Dealer (OH)	24%	27%	Yes	No bias	311,605
Dallas Morning News (TX)	13%	27%	Health	Conservative	409,265
Denver Post (CO)	14%	37%	Health, Tech	No bias	416,676
Honolulu Star-Advertiser (HI)	0%	39%	No	Conservative	268,244
Houston Chronicle (TX)	14%	27%	Yes (Blog)	No bias	360,251
Los Angeles Investor’s Business Daily (CA) ($) (*)	0%	41%	No	Not found	157,161
Los Angeles Times (CA)	15%	41%	Yes	Liberal	653,868
Miami Herald (FL)	10%	32%	Health	Liberal	147,130
Minneapolis Star Tribune (MN)	28%	34%	Yes	Liberal	301,345
New York Daily News (NY)	14%	39%	Health	No bias	516,165
New York Post (NY)	0%	39%	Yes	Conservative	500,521
New York Times (NY)	16%	N/A (National Scope)	Yes	Liberal	1,865,318
Newark Star-Ledger (NJ)	12%	38%	Health	Liberal	340,778
Newsday (NY)	0%	39%	Health	Liberal	377,744
Philadelphia Inquirer (PA)	33%	34%	Yes	Liberal	306,831
Riverside Co. Press-Enterprise (CA)	40%	41%	Health, Environment	Conservative	137,581
Salt Lake City Deseret News (UT)	22%	27%	Yes	Conservative	103,190
San Francisco Chronicle (CA)	29%	41%	Yes	Liberal	218,987
St. Paul Pioneer Press (MN)	13%	34%	Health	Liberal	208,280
USA Today (VA)	21%	N/A (National Scope)	Yes	Conservative	1,674,306
Wall Street Journal (NY) ($)	14%	N/A (International Scope)	Yes	Conservative	2,378,827

($) indicates financial specialization newspaper
(*) indicates publication on weekdays only; all other newspapers publish daily.

Percentage of articles using “evolve” based on political orientation

Political Orientation	Odds Ratio	95% Confidence Interval for Odds Ratio
Conservative vs. Liberal	1.042	(.704, 1.543)
Conservative vs. No Bias	1.008	(.598, 1.700)
Liberal vs. No Bias	0.968	(.601, 1.560)

Conclusion: No significant difference.

Percentage of articles using “evolve” based on science section

Science Section	Odds Ratio	95% Confidence Interval for Odds Ratio
Science vs. Health	1.33	(.887, 1.99)

Conclusion: No significant difference.

Correlation between percentage of articles using “evolve” and circulation:

P-value = 0.870

Conclusion: No significant correlation.

Correlation between percentage of articles using “evolve” and state view on evolution:

P-value = 0.565

Conclusion: No significant correlation.
